# Potato Protein Suppresses Proteolytic Activity and Improves Textural Property of Tropical and Cold-Water Fish Surimi

**DOI:** 10.3390/foods14193444

**Published:** 2025-10-08

**Authors:** Ali Hamzeh, Sunanta Chumee, Maarten Hotse Wilbrink, Robin Eric Jacobus Spelbrink, Marc Christiaan Laus, Jirawat Yongsawatdigul

**Affiliations:** 1 School of Food Technology, Institute of Agricultural Technology, Suranaree University of Technology, Nakhon Ratchasima 30000, Thailand; ahamze86@gmail.com (A.H.); sunanta.chumee@gmail.com (S.C.); 2 Avebe Innovation Center, Coöperatie Koninklijke Avebe U.A., Zernikelaan 8, 9747 AA Groningen, The Netherlands

**Keywords:** gel, breaking force, protease inhibition, proteolysis

## Abstract

Potato protein (PP) at concentrations of 0.025–0.3% was added to tropical fish surimi, including lizardfish (LZ) and threadfin bream (TB), and cold-water fish, namely Alaska pollock (AP) and Pacific whiting (PW), to examine its effect on proteolytic inhibition and surimi gel texture. Tropical fish surimi, particularly LZ, exhibited the highest degree of autolysis induced by endogenous proteases (*p* < 0.05), as evidenced by degradation of myosin heavy chain and tropomyosin. PP demonstrated a broad range of proteolytic inhibition activities against chymotrypsin, trypsin, papain, and cathepsin L, with chymotrypsin being the most susceptible. At a PP concentration of 0.3%, the highest autolytic inhibition was obtained in AP (72.24%), followed by LZ surimi (60.44%, *p* < 0.05). Egg white protein (EW) showed autolytic inhibitory activity at 14.50–50.52% in all species at 0.3%. Surimi gels with only 0.025% PP exhibited breaking forces and distance comparable to those with added 0.3% EW, regardless of the cooking regimes. In tropical surimi, PP at 0.3% increased the breaking force by 4.13–5.38-fold under a setting condition (as the best heating regime) compared with the control. At this concentration, PP decreased the whiteness of LZ and AP in the set surimi gels by 7.03% and 6.42% (*p* < 0.05), respectively, whereas its effect on TB and PW surimi was negligible. The study demonstrates that PP can be a promising alternative to EW to control proteolytic degradation and improve textural properties of cold-water and tropical surimi.

## 1. Introduction

Gel formation is an important factor in surimi seafood products, which plays a key role in their quality and marketability. Sol–gel transformation is generally induced by heat treatment, by which the protein is primarily denatured and the reactive moieties exposed. The unfolded protein is then rearranged and forms a network by intermolecular and intramolecular forces. However, the heat treatment can activate endogenous proteases generally with optimum temperatures of 40–65 °C, depending on fish species, which would exert destructive effects on the gel texture [1]. Serine proteases in threadfin bream (TB), cathepsins in Pacific whiting (PW), serine and cysteine proteases in lizardfish (LZ), and serine and cysteine proteases along with metalloproteases in Alaska pollock (AP) have been reported in the literature [2,3,4]. Although surimi production can wash out proteases to some extent, it cannot thoroughly eliminate them. An et al. [5] reported that cathepsins B and H were eliminated from PW mince through washing; however, cathepsin L remained and caused proteolysis during heat-induced gel formation. Rawdkuen and Benjakul [6] reported that serine and cysteine proteases are responsible for protein degradation in washed LZ mince. To this regard, protease inhibitors are generally applied in surimi to control endogenous proteolytic activity and textural breakdown.

Protease inhibitors from various animal and plant sources have been applied in surimi, among which egg white (EW) is currently used in most commercial surimi. However, egg is included as an allergen and may become contaminated with human pathogens, such as *Salmonella*. Plant-based protease inhibitors with a comparable inhibitory activity could be appropriate substitutions. Potato protein (PP) possesses unique features, including high biological value of 90 (100 for egg protein, 84 for soybean, and 73 for beans), less allergic responses, and 40–50% protease inhibition [3,7,8]. These features make it one of the effective plant-based additives with potential application in surimi. Protease inhibitors from potato tubers are a diverse group of molecules with inhibitory activities against cysteine, serine, and aspartic proteases as well as carboxypeptidases. When analyzed on reducing sodium dodecyl sulfate–polyacrylamide gel electrophoresis (SDS-PAGE), they occur as patterns of three major bands at 16–18, 20–22, and 30–32 kDa [9,10]. The secondary structure is mainly of the β-II type, i.e., antiparallel β-sheets and β-turns [11].

Few studies have investigated the effect of PP as a protease inhibitor on surimi gel properties. Yoon et al. [3] precipitated PP from potato juice using ethanol and added it to PW surimi. The ethanol-extracted PP exerted a proteolytic inhibitory effect on the PW surimi and improved its gel properties. The inclusion of potato extract was also found to be beneficial to the texture of PW and arrowtooth flounder surimi [12]. Waglay et al. [13] reported that the proteolytic inhibitory activity and yield of PP are affected by the extraction method. For example, ethanolic extraction demonstrated lower yield and higher inhibitory activity against trypsin than the acidic procedure. Furthermore, PP prepared using the acidic procedure exhibited higher activity against chymotrypsin. Application of PP in cold-water fish has been studied in limited context. Thus, systematic evaluation across both tropical and cold-water surimi should be investigated. Owing to the different endogenous proteases among fish species and surimi, the inhibitory activity of PP should be investigated in individual fish species. Evaluation of the PP effect in surimi from different fish species can facilitate comprehensive assessment of the worldwide application of PP in surimi industries.

PP extraction through mild processing allows the use of native PPs in various food systems for their techno-functional properties, such as gelling, foaming, and emulsifying properties. Protease inhibitors present in potato tubers possess biochemical properties that differ from other proteins present in tubers, facilitating the extraction of this specific protein group [14]. PP is considered as a non-allergenic protein ingredient and Generally Recognized as Safe (GRAS) according to the US Food and Drug Administration (FDA) [8]. Thus, the use of PP to replace EW in surimi would be industrially attractive regarding the allergenicity. However, technical aspects, especially the effect on protease inhibition and textural improvement, have not been systematically evaluated.

This study, thus, aimed to assess the proteolytic inhibitory effect of the commercially available mildly processed PP in surimi from tropical and cold-water fish species under different heat treatments, including low-temperature setting at 25 °C/40 °C followed by 90 °C, high-temperature setting at 65 °C (modori), and direct cooking at 90 °C.

## 2. Materials and Methods

### 2.1. Samples

Medium-grade PW surimi was obtained from Premier Pacific Seafoods (Seattle, WA, USA) and low-grade surimi AP surimi from UniSea Inc. (Redmond, WA, USA). Medium-grade LZ and TB surimi samples were purchased from Andaman Surimi Industries Co., Ltd. (Samut Sakhon, Thailand). The PW and AP contained 4% sucrose and 4% sorbitol, while LZ and TB were mixed with 6% sucrose as cryoprotectants. All surimi samples contained 0.3% mixed phosphates (sodium pyrophosphate and sodium tripolyphosphate). Blocks (1 kg) of each surimi were prepared, vacuum-sealed in plastic bags, and stored at −20 °C until further use. All surimi samples did not contain EW. PP (PerfectaMAR^®^ S400) with protein content of 82.4% manufactured according to the method of Giuseppin et al. [15] was obtained from Avebe (Veendam, The Netherlands). EW powder containing 60.8% protein was purchased from Igreca (Seiches-sur-le-Loir, France).

### 2.2. Inhibition Against Various Proteases

The inhibitory activity of PP and EW against trypsin, chymotrypsin, papain and cathepsin L was investigated. The inhibitory activity of trypsin was essentially determined as described by the International Standard ISO 14902:2001 method [16], adapted for measurement in a 96-well plate reader (Thermo Scientific Multiskan GO, Vantaa, Finland) using the synthetic substrate L-BAPA (Na-benzoyl-L-arginine-4-nitroanilide, Sigma-Aldrich B3279, Saint Louis, MO, USA) and trypsin (Sigma-Aldrich T9201). The activity toward chymotrypsin was assessed using the same ISO method with chymotrypsin (Sigma-Aldrich C4129). Reactions were carried out at 37 °C for 15 min in 200 µL final volume per well, consisting of 100 µL L-BAPA in buffer pH 8.2 (1.4 mmol/L L-BAPA in 50 mmol/L Tris-HCl + 5 mmol/L CaCl_2_ and 1% DMSO), 20 µL sample (or water for control), 20 µL protease (trypsin 27 µg/mL, chymotrypsin 135 µg/mL), and 60 µL deionized H_2_O. The absorbance at 410 nm was read with 15 sec intervals and enzyme activities were determined through linear regression. The inhibitory activity of papain (Merck-Millipore 1.07144, Merck & Co., Rahway, NJ, USA) was determined using the aforementioned method with the same concentration of L-BAPA as a substrate, which was dissolved in buffer at a pH of 6.5 (100 mmol/L K_2_HPO_4_, 300 mmol/L KCl, 4 mmol/L EDTA, and 16 mmol/L L-cysteine and 1% DMSO), in a total reaction volume of 200 µL (similar to above), using 20 µL papain (135 µg/mL). Cathepsin L inhibition was determined using the InnoZyme Cathepsin L Activity Kit (Millipore CBA023) and a BioTek Synergy HTX reader (Agilent Technologies, Santa Clara, CA, USA) at λex = 360 and λem = 460 nm at 37 °C according to the manufacturer’s protocols.

### 2.3. Autolytic Activity and Proteolytic Inhibition in Surimi

Autolytic activity in surimi and proteolytic inhibition capability of PP and EW in surimi were determined according to Tadpitchayangkoon et al. [17]. Surimi (3 g) was mixed with PP or EW at various concentrations of 0%, 0.025%, 0.05%, 0.1%, 0.2%, and 0.3% (*w*/*w*). The mixture was incubated at 55 °C and 65 °C for 60 min. Subsequently, 27 mL of 5% cold trichloroacetic acid was added to the sample and homogenized at 10,000 rpm for 1 min. The homogenate was allowed to precipitate at 4 °C for 60 min and then centrifuged at 8000× *g* for 10 min. The content of produced oligopeptides was quantified using the method of Lowry et al. [18] with L-tyrosine as the standard based on the following equation:

Inhibition (%) = ((TC_t_ − TC_0_) − (TI_t_ − TI_0_))/((TC_t_ − TC_0_)) × 100 (1)

where TC_t_ denotes the control sample incubated at either 55 °C or 65 °C; TC_0_, the control sample placed in ice; TI_t_, the sample treated with inhibitors and incubated at 55 °C or 65 °C; and TI_0_, the sample treated with inhibitors placed in ice.

### 2.4. Surimi Gel Preparation

Surimi samples with known moisture content were chopped and mixed using a vacuum silent cutter (UM 5Universal, Stephan U. Söhne GmbH & Co., Hameln, Germany). Sodium chloride (2%, *w*/*w*), PP (0%, 0.025%, 0.05%, 0.1%, 0.2%, and 0.3%, *w*/*w*) or EW (0.3%, *w*/*w*), and ice, required to reach 78% moisture content as recommended by FAO/WHO [19], were added. The mixture was chopped at a low speed for 1 min with each ingredient addition. Finally, high-speed chopping for 3 min was applied, and surimi paste was stuffed into polyvinylidene chloride casings (3 cm diameter) using a vertical sausage stuffer. The surimi samples were divided into three groups and subjected to (1) setting for 2 h at 25 °C for PW and AP or 30 min at 40 °C for LZ and TB, followed by 30 min cooking at 90 °C; (2) 30 min preincubation at 65 °C, followed by 30 min cooking at 90 °C; and (3) direct cooking at 90 °C for 30 min. After thermal treatment, the gels were cooled in ice water for 30 min and stored at 4 °C overnight before further analysis.

### 2.5. Textural Properties

Surimi gels (3 cm height and diameter) were tested for gel strength using TA-XT plus Texture Analyzer (Stable Micro Systems Ltd., Surrey, UK) with a 5 mm spherical probe (P/5S) as described by Pao et al. [20]. A 25 kg loading cell was used, with a probe speed rate of 1 mm/s. At least 10 measured values were obtained for breaking force (g) and breaking distance (mm).

### 2.6. Color

The color indices (L*, a*, b*) of surimi gel cuts (3 cm × 3 cm) were measured in six replicates using a ColorQuest XE colorimeter (Hunter Associates Laboratory, Inc., Reston, VA, USA) with daylight (D65) illumination in the reflectance mode. The colorimeter was calibrated with a white ceramic standard before each use [21]. The results were reported in the CIE color system, and whiteness was calculated as follows:

Whiteness (%) = L* − (3 b*) (2)

### 2.7. Water-Holding Capacity

Surimi samples (0.5 cm, ~3 g) were weighed and placed in centrifuge tubes using a double-layered Whatman No. 1 filter paper. The samples were centrifuged at 2000× *g* for 10 min. The weight of the samples before and after centrifugation without a filter paper was recorded, and the water-holding capacity (WHC) was calculated as follows [22]:

(3) $$WHC\;(\%)=\frac{W_{2}}{W_{1}}\times 100$$

where W_2_ denotes the weight of the sample after centrifugation and W_1_ denotes the initial weight of the sample.

### 2.8. Sodium Dodecyl Sulfate–Polyacrylamide Gel Electrophoresis (SDS-PAGE)

Protein patterns were analyzed via SDS-PAGE, according to the method of Laemmli [23], with slight modifications. A sample (1 g) was dissolved in 10 mL of hot 10% SDS solution. Subsequently, the mixture was homogenized at 10,000 rpm for 2 min and then heated at 90 °C for 15 min. After cooling to room temperature, the sample was centrifuged at 8000× *g* for 20 min at 25 °C. Protein content was analyzed using the Lowry method [18]. The protein solution was then mixed with treatment buffer (0.125 M Tris-HCl, 4% SDS, 20% glycerol, and 10% β-mercaptoethanol, pH 6.8) in a 1:1 (*v*/*v*) ratio and heated to 95 °C for 5 min. An aliquot of 20 µL protein samples (20 µg/well) was separated using 10% polyacrylamide gel and 4% stacking gel under 100 V (Bio-Rad Laboratories, Inc., Hercules, CA, USA). A standard marker with the molecular range of 250–10 kDa (Bio-Rad Laboratories, Inc., CA, USA) was used. Proteins were stained with Coomassie Brilliant Blue R-250 in 40% methanol and 10% acetic acid for 1 h and destained with a solution consisting of 50% methanol and 10% acetic acid until clear protein bands were visible. The gel was photographed using the UviTec system (Bio-Active Con., Ltd., Cambridge, UK).

### 2.9. Statistical Analysis

Experiments were conducted at least in triplicate, and statistical analysis was conducted using SPSS 16.0 (SPSS Inc., Chicago, IL, USA). Analysis of variance was conducted at a 95% confidence level, and means were compared using Duncan’s new multiple range test.

## 3. Results and Discussion

### 3.1. Proteolytic Inhibitory Activity

**Figure 1:**
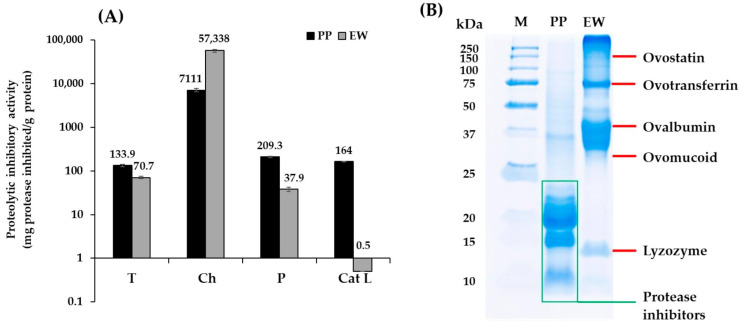
(**A**): Proteolytic inhibitory activity (mg protease inhibited/g protein) of potato protein (PP) and egg white (EW) against various proteases. T: Trypsin. Ch: Chymotrypsin. P: Papain. Cat L: Cathepsin L. (**B**): Protein pattern of PP and EW. M: Protein standard markers.

Serine and cysteine protease activities play a role in the deterioration of surimi texture. The in vitro proteolytic inhibitory activity of PP and EW against serine proteases (trypsin and chymotrypsin) and cysteine proteases (papain and cathepsin L) were determined using synthetic substrates (Figure 1A).

PP demonstrated a broad range of proteolytic inhibitory activities, with the highest activity shown against chymotrypsin as well as papain and cathepsin L. Such a broad-spectrum inhibitory activity is in agreement with the result observed by Pouvreau et al. [9] who characterized the different protease inhibitors present in potato. The authors reported that protease inhibitors in potato protein had molecular weight of lower than 25 kDa (approximately 20–22, 8 and 4 kDa). Cysteine and serine protease inhibitors were reported to be the 20–22 kDa fraction, known as protease type II; the 8 kDa fraction included class I protease inhibitors (such as serine protease inhibitor), while carboxypeptidase inhibitor was only present in the 4 kDa fraction. Similarly, Katzav et al. [24] reported that protease inhibitors in PP were in the range of 5–25 kDa on SDS-PAGE. Potato protease inhibitors are reported to act as canonical inhibitors with competitive inhibition mode, which bind to the active site of protease [25]. The class II potato protease inhibitors (~20 kDa) possess two inhibiting domains, enabling them to inhibit two proteases at the same time. Domain 1 has been reported to act as a typical inhibitor against trypsin and chymotrypsin, while the second domain is a versatile inhibitor [26]. The major bands of PP in this study have been observed to have a molecular weight lower than 25 kDa (Figure 1B). A band with a molecular weight of about 37 kDa corresponded to patatin, while other bands with molecular weights of about 18–22 and 8 kDa corresponded to previously reported class II and I protease inhibitors, respectively.

EW demonstrated inhibitory activity against serine protease, with the highest activity shown against chymotrypsin. Ovostatin and ovomucoid with low intensities were major proteins possessing protease inhibitory activity (Figure 1B), which mainly act as serine protease inhibitors, particularly ovomucoid [27]. This may explain the lower inhibitory activity observed in EW against the tested cysteine proteases (papain and cathepsin L) compared with that obtained using PP. Ovomucoid has been reported to hinder trypsin activity through hydrogen binding formation of its first domain in a non-competitive mode of action [28]. Trypsin-like proteases have been identified from the skeletal muscles of crucian carp (*Carassius auratus*), grass carp (*Ctenopharyngodon idella*), and Argentine hake (*Merluccius hubbsi*), which play roles in their muscle degradation [29,30,31].

### 3.2. Autolytic Activity

All the studied commercial surimi exhibited high proteolytic activity at 55 °C and 65 °C (Table 1), among which LZ showed the highest value among the fish species, followed by TB, AP and PW, respectively (*p* < 0.05). The greatest activity was observed in LZ at 65 °C (*p* < 0.05), whereas that of TB was observed at 55 °C (*p* < 0.05). LZ surimi is well known for its high proteolytic activity in muscles, and previous studies have reported its distinct characteristic as one of the protease-laden tropical surimi [4,17].

**Figure 2:**
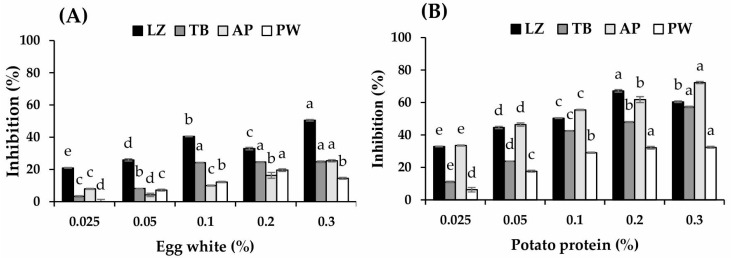
Proteolytic inhibition of egg white (**A**) and potato protein (**B**) at concentrations of 0.025–0.3%. LZ, lizardfish surimi; TB, threadfin bream surimi; AP, Alaska pollock surimi; PW, Pacific whiting surimi. Different letters indicate the differences in each surimi at different concentrations of protease inhibitor (*p* < 0.05).

The proteolytic inhibitory activity of PP varied with fish species and was higher than that of EW (*p* < 0.05, Figure 2A,B). The highest inhibitory activity of PP was observed in AP (72.24%), followed by LZ and TB (67.2% and 57.3%, respectively), at 0.2–0.3% PP (*p* < 0.05). A lower efficacy of PP was observed in PW with an inhibition of 32.4%. EW showed higher efficacy in tropical fish surimi samples (LZ and TB) than cold-water species (AP and PW). However, the inhibitory activity of EW was much lower than that of PP at concentrations of up to 0.3%. Serine protease is a major activity type responsible for the textural breakdown of TB and LZ [4,32]. Ovomucoid with an approximate proportion of 11% in EW protein is the main protease inhibitor in EW, which has been reported to act as a serine protease inhibitor [27]. This could explain the higher efficacy of EW in tropical fish surimi. In contrast, protease inhibitors make up 45–50% of PP involving various inhibitor classes against cysteine, serine, and aspartate proteases as well as metallocarboxypeptidase [33]. This would result in higher proteolytic inhibitory activity of PP.

### 3.3. Gel Texture

**Figure 3:**
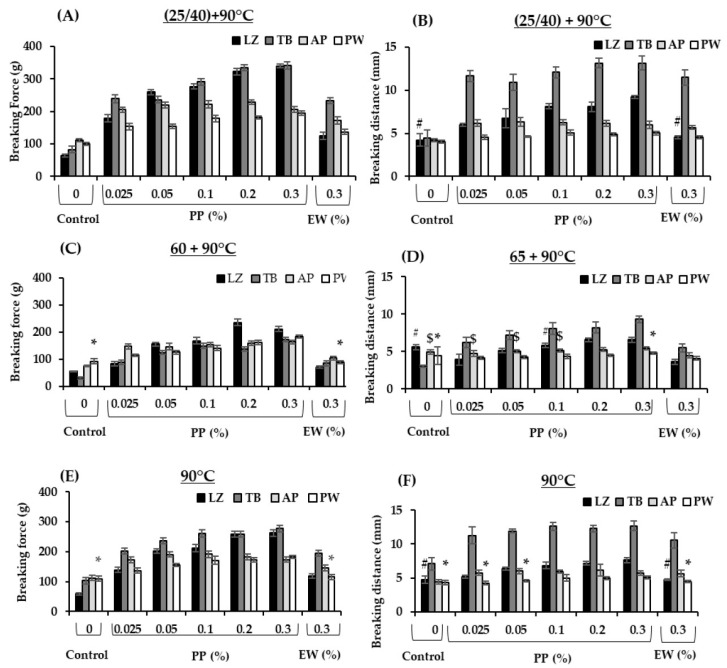
The breaking force (**A**,**C**,**E**) and breaking distance (**B**,**D**,**F**) values of surimi gels formulated with potato protein (PP) and egg white (EW) prepared in various heating regimes. LZ, lizardfish surimi; TB, threadfin bream surimi; AP, Alaska pollock surimi; PW, Pacific whiting surimi. Setting of LZ and TB was carried out at 40 °C, whereas that of AP and PW was at 25 °C. Those with comparable values to the corresponding control group are marked as # in lizardfish surimi, $ in Alaka pollock surimi and * in Pacific whiting surimi (*p* > 0.05).

The textural properties of surimi gel samples were evaluated at various thermal treatments (Figure 3A–F). EW at 0.3% was used as a positive control as this concentration has been widely used in surimi industries to control endogenous proteases in frozen surimi. Gel improvement at setting was modest in surimi samples without protein inhibitor (Figure 3A,B). The optimal setting condition of tropical surimi has been reported to be 40 °C, whereas that of cold and temperate species was 25 °C [34,35,36]. Proteolytic activity at 40 °C is likely to occur, resulting in textural degradation at 40 °C in LZ and TB when neither EW nor PP was incorporated. In contrast, setting enhanced the gel properties of surimi with added protease inhibitors, particularly those with added PP. The breaking force and distance of LZ and TB surimi increased by approximately 4–5 and 2–3 times, respectively, when 0.3% PP was added, whereas about a 1.8-fold increase in breaking force and 1–2.5 times increase in breaking distance were observed in AP and PW (Figure 3A,B). EW at 0.3% did not improve the breaking distance of LZ surimi under the setting condition. The gel enhancement efficacy of PP in the setting condition appeared to be more pronounced in these two tropical surimi species. Notably, the textural properties of four surimi samples under the setting condition with only 0.025% PP were comparable to those with added 0.3% EW, demonstrating the higher efficacy of gel enhancement under the setting condition of PP (Figure 3A,B). The higher inhibitory activity of PP likely minimized proteolytic degradation in the setting condition, which led to stronger gel networks. Pouvreau et al. [9] purified seven classes of protease inhibitors from potato juice, which constituted 50% of the total soluble proteins. Serine proteases followed by cysteine proteases with proportions of 22% and 12%, respectively, were the predominant inhibitors. The protease inhibitors showed broad-spectrum activity, particularly against trypsin and chymotrypsin, which agrees with the study (Figure 1A). Visser et al. [37] also observed that PP had the potential to inhibit cathepsin B, cathepsin L, metallocarboxypeptidase, trypsin, chymotrypsin, elastase, and ACE2. The higher effectiveness of PP compared with EW was also reported in the gel properties of PW surimi [3].

At 65 °C, textural improvement was observed in all samples with both protein additives (Figure 3C,D). An increase in the breaking force of all fish species was found when PP was added at a concentration as low as 0.025% and increased with the addition of PP. Notably, the breaking force and distance values of gels with added 0.025% PP were comparable to or higher than those added with 0.3% EW in all species. At the same addition of 0.3%, gels with added PP exhibited a 2–5.8-fold increase in the breaking force compared with the control (without additive), whereas only a 1.3–2.8-fold increase was observed in EW. The breaking distances in surimi with added PP also increased by 1–1.7-fold compared with those of surimi with added EW. A maximum improvement was observed in TB surimi gel with added PP that exhibited a 5.8-fold increase in breaking force and 2.6-fold increase in breaking distance compared with the control (without inhibitor). TB gels with added EW exhibited lower breaking distance than those with added PP at all concentrations. These results indicated that PP was more effective than EW in minimizing textural degradation under modori heat treatment. PW did not show any breaking force improvement with EW addition, whereas a twofold increase in breaking force was observed with PP addition. As cathepsin L is a major protease in PW, the enzyme would not be effectively inhibited by a serine protease inhibitor in EW. The LZ and AP gels were also improved by PP to a greater extent than by EW. Our results indicated that PP is more effective in minimizing the textural degradation of surimi subjected to modori induction. Tropical fish surimi appeared to exert greater effects on the gel improvement of PP. Serine proteases are mainly involved in the textural degradation of tropical surimi. This phenomenon could be partly explained by greater inhibition of serine proteases, particularly trypsin- and chymotrypsin-like proteases. In addition, Moreno et al. [38] and Luo et al. [39] reported that pea and soy protein isolates at concentrations of 1.4% and 10% improved the gel properties of AP and grass carp surimi, respectively. Black bean and mungbean protein isolates at concentrations of 0.25–1.5% improved gel texture through inhibition of autolytic activity [40]. Our study suggested that PP is more effective than EW as well as black bean, mungbean, soy and pea protein isolates in controlling the textural degradation of surimi gel at 65 °C at a concentration as low as 0.025%. This is mainly attributed to distinct protease inhibitors presenting in PP.

The effect of PP on surimi gel directly cooked at 90 °C was also evident compared with the control and those with added EW, particularly in tropical fish surimi (Figure 3E,F). At the same EW concentration of 0.3%, surimi with added PP showed 2.2 and 1.43 times higher breaking force as well as 1.6 and 1.2 times higher breaking distance in LZ and TB, respectively, compared with those with added EW. However, PP was more effective than EW at a concentration as low as 0.025%. All the other surimi samples with added 0.025% PP exhibited comparable or higher breaking force and breaking distance than samples with added 0.3% EW. This study demonstrates the effectiveness of PP in four surimi samples, particularly in tropical fish, upon various heating regimes.

### 3.4. Protein Patterns on SDS-PAGE

**Figure 4:**
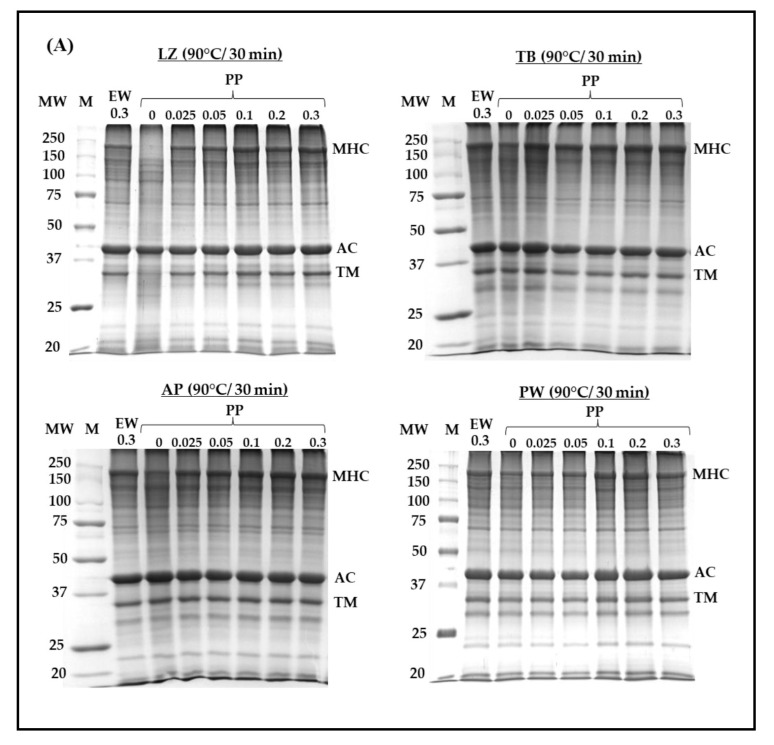

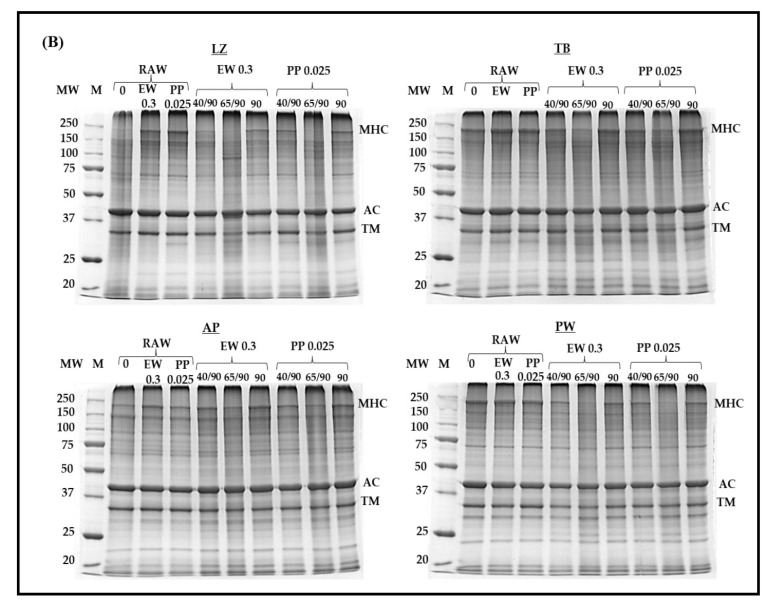
Sodium dodecyl sulfate–polyacrylamide gel electrophoresis (SDS-PAGE) of surimi from different fish species with different added concentrations of potato protein (**A**) and heat treatments (**B**). LZ, lizardfish surimi; TB, threadfin bream surimi; AP, Alaska pollock surimi; PW, Pacific whiting surimi; M, marker; PP, potato protein; EW, egg white; MHC, myosin heavy chain; AC, actin. TM, Tropomyosin. Protease inhibitors are in %.

The protein pattern of surimi gels prepared using different PP concentrations was assessed and compared with that obtained using EW at a concentration of 0.3% (Figure 4). Myosin heavy chain (MHC), actin, and tropomyosin were predominant. Among them, MHC, as the main part contributing to surimi gel formation, was affected upon the addition of protease inhibitors and heat treatment. MHC totally disappeared in LZ surimi without any protease inhibitors, even in the fast cooking regime (no incubation, Figure 4A). Moreover, the protein band corresponding to tropomyosin demonstrated the lowest intensity, and actin showed less susceptibility to proteolysis (Figure 4A). The proteolytic resistance of actin has been widely reported [6,41,42,43]. Rawdkuen and Benjakul [6] evaluated autolysis in tropical fish surimi and found that MHC and tropomyosin in LZ, goatfish, and TB surimi degraded upon autolysis, whereas their actin remained intact. LZ surimi without protease addition showed significant degradation of MHC and TM (Figure 4A). The results corresponded to the autolytic activity presented in Table 1, in which LZ surimi exhibited the greatest autolytic activity. Regardless of the fish species, the intensity of the MHC band increased as the PP concentration increased. Similar to EW, the inhibitory effect of PP against endogenous proteases resulted in greater MHC retention; however, PP was more effective. In LZ surimi, the MHC intensity of samples with added 0.3% EW was comparable to that with added 0.025% PP. MHC autolysis was also observed in other surimi samples but to a lower extent than LZ surimi. The intensity of the MHC band in PW surimi was apparently higher than those in other samples, which indicated lower autolysis, as presented in Table 1. Due to the comparable effect of 0.3% EW to 0.025% PP, surimi samples with such added concentrations of the two inhibitors were subjected to different heat treatments (Figure 4B). Raw LZ surimi paste exhibited severe MHC degradation when no protein inhibitor was added, suggesting substantial proteolysis during surimi paste preparation, although the temperature of the whole process (surimi cutting, chopping, stuffing) was controlled below 15 °C. Addition of 0.3% EW or 0.025% PP greatly protect MHC loss induced by endogenous proteases (Figure 4B). LZ demonstrated more severe autolysis at 40 °C and 65 °C even in the presence of both inhibitors, in which tropomyosin also degraded in the latter. Such degradations also occurred in TB surimi but to a lower extent. However, it should be noted that the lower intensity of MHC in protease-laden species at 40 °C might be related to the concurrence of autolysis and transglutaminase-induced crosslinking [44]. AP surimi showed lower MHC intensity both with and without protease inhibitors. MHC degradation was also observed in PW surimi at 65 °C, and both inhibitors had limited protective effects towards MHC, although PP was applied at a concentration 10 times lower than that of EW. Based on these results, the improvement in gel strength observed in all surimi samples with 0.025% added PP was primarily attributed to the reduction in MHC proteolysis. It is unlikely that gelation of PP contributed to strengthening surimi gels, as the applied concentration (0.025%) was probably below the critical level required for its own gelation.

However, further studies on the functional properties of PP—particularly its gelation behavior—are needed to better understand the mechanisms behind its gel-enhancing effects beyond its role as a protease inhibitor.

### 3.5. Water-Holding Capacity (WHC)

**Figure 5:**
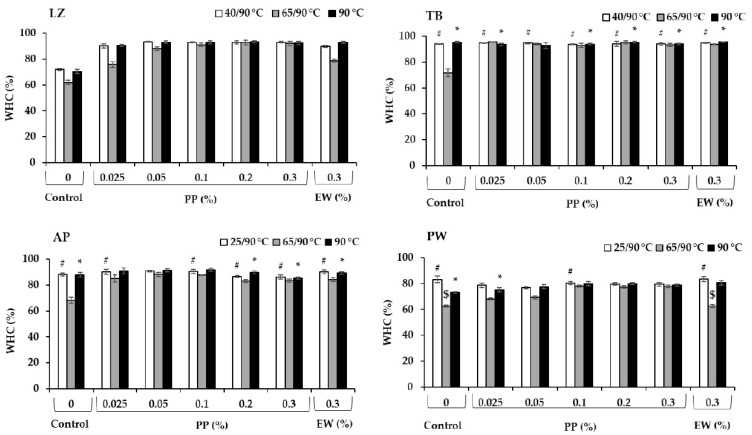
Water-holding capacity (WHC, %) of surimi gels prepared from lizardfish (LZ), threadfin bream (TB), Alaska pollock (AP), and Pacific whiting (PW) with added egg white (EW, 0.3%) and potato protein (PP, 0.025–0.3%) along with control (no protease inhibitor) in three different heat treatments. Those with comparable values with the corresponding control group are marked as # in low-temperature-set gels, $ in modori gels and * in directly cooked gels (*p* > 0.05).

Among various surimi samples without protease inhibitors (controls), LZ exhibited the lowest WHC in all heat treatments (Figure 5), which was likely due to the greatest autolytic activity (Table 1). However, the different protein intrinsic features (structure, conformation and non-polar and polar amino acid residues) among different fish species would also have effects on the water retention [45]. Expectedly, modori gels without protease inhibitors exhibited the lowest WHC, reflecting poor gel networks. The severe MHC degradation observed in Figure 4 hampered strong gel network formation, leading to a lower water retention capability. A higher expressible moisture content in modori gel compared with kamaboko gel from sardine was also reported, in which addition of 0.25–1.5% black bean and mungbean protein isolates improved water retention ability of the gels through proteolytic inhibition as indicated by reducing TCA-soluble oligopeptide contents [40]. The addition of protease inhibitors, either EW or PP, increased the WHC of gels subjected to various heating regimes with different efficacies. PP was more effective in increasing the WHC of gels prepared by setting and direct cooking (90 °C) compared with EW, as a lower concentration of 0.025% PP was successfully applied in LZ, TB, and AP. However, PP’s effect on the WHC of PW surimi was not promising compared with those of other fish species used in this study as the addition of 0.1% PP to PW surimi gel prepared by direct cooking exerted a comparable effect to 0.3% EW (*p* > 0.05), and the addition to the set gel exerted a deteriorating effect (*p* < 0.05). This was in accordance with the lower autolytic inhibition capacity of PP in PW (Figure 2). PP exerted a pronounced effect on the improvement of the WHC of the modori gels of LZ, TB, and PW, enabling them to reach a comparable WHC to gels prepared by setting and direct cooking (90 °C) at 0.1%, 0.025%, and 0.3% concentrations, respectively. In contrast, only the modori gel of TB with added EW reached a comparable WHC to the corresponding gels prepared at 40 °C/90 °C and 90 °C. The EW in TB also showed high efficacy in preventing autolytic activity.

### 3.6. Whiteness

**Figure 6:**
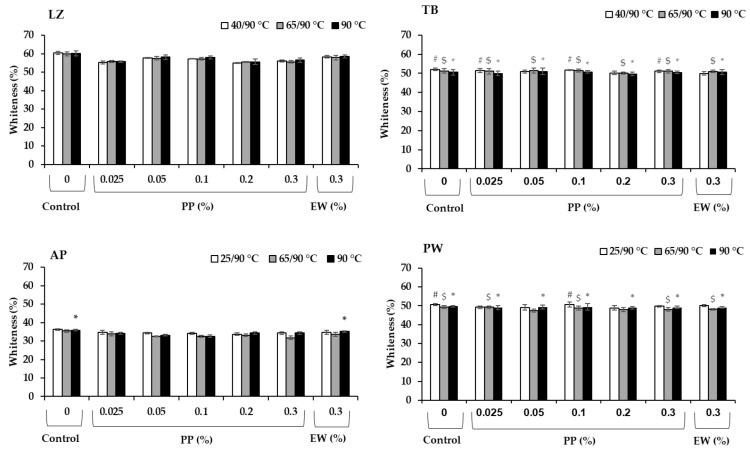
Whiteness of surimi gels prepared from lizardfish (LZ), threadfin bream (TB), Alaska pollock (AP), and Pacific whiting (PW) with added egg white (EW, 0.3%) and potato protein (PP, 0.025–0.3%) along with control (no protease inhibitor) in three different heat treatments. Those with comparable values to the corresponding control group are marked as # in low-temperature-set gels, $ in modori gels and * in directly cooked gels (*p* > 0.05).

The whiteness values of the different surimi samples are presented in Figure 6. Generally, whiteness correlated to the fish species, in which LZ had the highest value (*p* < 0.05), followed by TB and PW, whereas the recovery-grade AP exhibited the lowest value (*p* < 0.05). PP exerted a considerable effect on LZ and AP surimi gels, in which the whiteness reduced with the addition of PP, with the reduction higher than that of EW. At the same concentration of 0.3%, PP reduced the whiteness in LZ and AP by approximately 6–7% and 4–10% (depending on the heating regimes), respectively, whereas the values were in the ranges of 2–3% and 2–5% in samples with added EW. Such a whiteness reduction in surimi was also reported in plant-based protease inhibitors including protein isolates from black bean, mungbean, and coconut husk extract [40,46], which is correlated to the inherent color of their sources. The PP used in this study had a yellowish color. The highest whiteness value in the surimi samples was achieved in the control (*p* < 0.05). The control samples in TB and PW surimi without protease inhibitors showed a slightly higher whiteness, but not significant (*p* > 0.05). These results indicated that the effect of PP on whiteness reduction of surimi from TB and PW is negligible.

## 4. Conclusions

PP showed broad-range inhibitory activity against serine and cysteine proteases, particularly chymotrypsin and cathepsin L. It also demonstrated effective proteolytic inhibitory activity against endogenous proteases in surimi prepared from four fish species, namely, two tropical fish (medium-grade LZ and TB) and two cold-water fish (low-grade AP and medium-grade PW). Surimi containing 0.025% PP showed higher or comparable textural properties (breaking force and distance) and WHC to those containing 0.3% EW in three different heating regimes. PP at only 0.025% is more effective in minimizing proteolytic and textural degradation in tropical fish surimi, which has greater proteolytic activity than cold-water fish. These results illustrated the scientific support for PP as a promising protease inhibitor substitute for EW in surimi on an industrial scale. However, the effects of PP on surimi seafood products can expand its potential application in industry, in which knowledge on its capability in different environments regarding salt concentration, pH, and wide temperature ranges, including sterilization, would be beneficial.

## Figures and Tables

**Table 1:** TCA-soluble oligopeptide content (mg/g sample) of various surimi samples incubated at 55 and 65 °C for 60 min.

Sample	Control	55 °C	65 °C
LZ	1.05 ± 0.02 ^Ca^	6.18 ± 0.03 ^Ba^	7.67 ± 0.02 ^Aa^
TB	0.43 ± 0.00 ^Cb^	3.44 ± 0.03 ^Ab^	2.67 ± 0.07 ^Bb^
AP	0.34 ± 0.00 ^Cc^	1.14 ± 0.00 ^Bc^	1.24 ± 0.00 ^Ac^
PW	0.46 ± 0.01 ^Cb^	1.09 ± 0.02 ^Bd^	1.15 ± 0.02 ^Ad^

LZ, lizardfish; TB, threadfin bream; AP, Alaska pollock; PW, Pacific whiting. Different uppercase and lowercase superscripts indicate difference within the row and column, respectively (*p* < 0.05).

## Data Availability

The raw data supporting the conclusions of this article will be made available by the authors on request.
